# Genotype by Trait Interaction (GT) in Maize Hybrids on Complete Fertilizer

**DOI:** 10.3390/plants10112388

**Published:** 2021-11-05

**Authors:** Seyed Mohammad Nasir Mousavi, Csaba Bojtor, Árpád Illés, János Nagy

**Affiliations:** Institute of Land Use, Engineering and Precision Farming Technology, Faculty of Agricultural and Food Sciences and Environmental Management, University of Debrecen, 138 Böszörményi St., H-4032 Debrecen, Hungary; bojtor.csaba@agr.unideb.hu (C.B.); illes.arpad@agr.unideb.hu (Á.I.); nagyjanos@agr.unideb.hu (J.N.)

**Keywords:** maize, GGE biplot, AMMI analysis, NPK fertilizer

## Abstract

We investigated the interaction between genotype by trait, and an experiment was conducted at the University of Debrecen. Two maize cultivars, FAO340 and FAO410, were studied in a randomized complete block design with four replications. This experiment was applied to the six fertilization treatments. Fertilizer levels were NPK0 (control) (N:0, P2O5:0, K2O:0), NPK1 (N:30, P2O5:23, K2O:27), NPK2 (N:60, P2O5:46, K2O:54), NPK3 (N:90, P2O5:69, K2O:81), NPK4 (N:120, P2O5:92, K2O:108), and NPK5 (N:150, P2O5:115, K2O:135). The first principal component showed 54.24%, and the second principal component showed 20.75%, which explained the total squares interaction using the AMMI model in the case of the FAO410 hybrid. As regards the FAO340 hybrid, the first principal component showed 58.18%, and the second principal component showed 18.04%, explaining the total squares interaction using the AMMI model in the FAO410 hybrid. In the GGE biplot on FAO410, the first and the second principal components covered 91.20% of the total data in this analysis. Accordingly, the desirable treatment was NPK5, followed by NPK4, NPK2, NPK3, NPK1, and NPK0. NPK4 and NPK5 had the most desirable treatments for the number of seeds per row, chlorophyll, weight of 1000 seeds, and stem diameter in the case of the FAO410 hybrid.

## 1. Introduction

Maize is the first crop in production and the second crop after wheat in the crop area. Maize is mostly used and traded for animal nutrition purposes, but it is also an integral part of the human food basket [1]. Although the results of all experiments have not been in agreement with each other, in most experiments, some parameters such as photosynthesis, leaf count, node count, chlorophyll content, plant height, total seed weight, ear length, seed number per cob, seed number in column, outer ear diameter, seed number in row, weight of cob corn, stem diameter, 1000 seeds in fresh and dry weight, oil content, protein, and starch content have great importance in determining yield. The relationship between traits in the case of the different genotypes can be an acceptable result in a few years for more accurate analysis than the existing conditions for achieving the goal to gain a decent yield [2,3,4,5,6,7,8,9]. Nitrogen is the most important element in soil fertility in the main axis of fertilizers. Phosphorus is needed for the crop to ripen quickly and prevent the plant from being damaged during the pollination stage. Potassium increases the length of the grain-filling period and helps to increase the number of grains in the ear [10].

GT or genotype by traits biplot by GGE biplot technique is used to study effect traits on genotypes. GGE is an excellent biplot to show interaction [11]. GT biplot can be used to learn genetics correlation [12,13,14,15], as used in a variety of evaluations of soybean [16], white lupin [14], bean [17], and oat [15,18,19]. As plant breeders increasingly use biplots, the correct interpretation of GT biplots becomes essential. Visualizing marker attributes, GT biplot can draw from origin to traits. The angle between the two traits can make a correlation coefficient. All biplots presented in this study were generated using the GGEbiplot software or Genstat Software [20]. The GT biplot method is used as one of the GPL biplot methods for genotype analysis by trait data. This study showed that GT biplot is an excellent tool for genotype identification in terms of trait interaction. The GGE biplot technique is used to assess the relationship between traits by two genotype–trait diagrams. A similar study showed that GT biplots are an excellent way to show interrelationships between traits [12,17,18,21]. The GT can also provide a visual comparison among genotypes by traits [13,14,15,16]. A study of 25 forage maize plants concluded that the GGE biplot method with different perspectives could be reliably used in evaluating the forage characteristics of corn hybrids grown in different conditions. The correlation of genotype by traits on chickpea cultivars concluded that most traits positively and significantly correlate with grain yield traits [22]. The need to increase domestic production and prevent a decline in maize yield has led farmers to use a variety of inputs of pesticides and chemical fertilizers without considering the immediate and long-term effects on the environment. Sustainable agriculture relies on methods in which pesticides and chemical fertilizers are used sparingly and optimally. While observing these principles, the concept of sustainable agriculture in agriculture refers to a set and range of activities such as integrated pest management and the use of special agricultural management techniques, which also leads to the stability of the agricultural system [23]. This study’s objective was to evaluate yield components of the genotypes by the trait interaction effect on different fertilizer levels in long-term experiments.

## 2. Materials and Methods

### 2.1. Site Description and Experimental Design

An experiment was conducted at the Faculty of Agriculture research farm, University of Debrecen, to study of the interaction between genotypes and traits. The experiments were carried out at the Látókép Experimental Station of the University of Debrecen (47°83′30 N, 21°82′60 E, 111 m a.s.l.). Two maize cultivars, FAO340 (Sushi Hybrid) and FAO410 (SY Premeo Hybrid), were studied in this experiment, which had a randomized complete block design with four replications. Plant spacing was selected as 20 cm. Seeds were disinfected before planting. In this experiment, the genotypes were sown with a kernel number of 72,000 plants/ha, applied to the six fertilization treatments. Fertilizer treatments were NPK0 (control) (N:0, P2O5:0, K2O:0), NPK1 (N:30, P2O5:23, K2O:27), NPK2 (N:60, P2O5:46, K2O:54), NPK3 (N:90, P2O5:69, K2O:81), NPK4 (N:120, P2O5:92, K2O:108), and NPK5 (N:150, P2O5:115, K2O:135). The phosphorus and potassium fertilizer were applied before winter plowing, and the nitrogen fertilizer was applied in the spring before the sowing begins. This study is part of a 38-year-old multifactorial fertilization field experiment. In this evaluation, the experimental plot was at the University of Debrecen. Our experiment was carried out at Látókép in 2018–2020. Each hybrid included 24 plots (six treatments in four replications). The station is in Eastern Hungary, 15 km from Debrecen in the Hajdúság loess region, and its soil is calcareous chernozem soil. The experimental soil was medium-hard loam. Its humus content is medium at 2.8%, and its pH value is almost neutral, pHKCl = 6.2. Continental and often extreme conditions characterize the climatic-meteorological factors of the experimental area; the soil is calcareous chernozem with 80–90 cm depth topsoil and 2.7 Hu% humus. The soil has a pH of 6.6 (slightly acidic). In terms of its physical variety, it is a clayey loam with a plasticity index of KA 44, according to Arany. All methods were performed in accordance with the relevant guidelines/regulations/legislation.

### 2.2. Climate Condition from Látókép in 2018–2020

**Figure 1:**
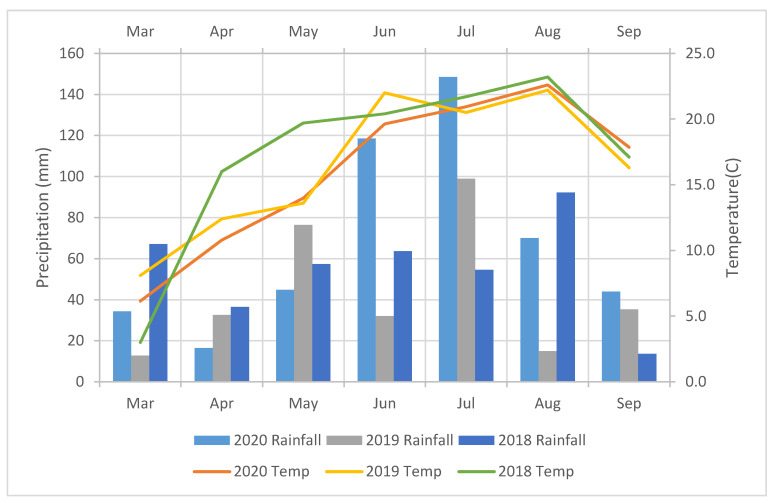
Monthly mean temperature and precipitation in 2018–2020 in Debrecen.

Local measurements determine the daily precipitation sum. The daily radiation and temperature data were provided by the Debrecen University’s agricultural monitoring center in the case of planting without irrigation and under rain-fed conditions. Among the various agrometeorological parameters, an analysis was made of the precipitation during the growing season. Sowing was carried out on 24th in April in the long-term experiment in 2018–2020. The daily rainfall sum is specified on local measurements. The total rainfall from May until October was 291 mm in 2018, 279 mm in 2019, and 482 mm in 2020. During the growing season, there were favorable conditions for maize production, including precipitation and temperature. In April, the somewhat dry and warm climate had a desirable impact. However, there was nearly average precipitation from April until May (average 93.9 mm) due to the dried seedbed condition. There was not any problem with germination because of the excellent condition of soil and the precipitation. There was favorable precipitation and temperature during the growing season, and ideal conditions were provided for maize development, growth, and yield formation (Figure 1).

### 2.3. Statistical Analysis

In this research, traits include chlorophyll content (Chl) (measuring leaf chlorophyll content with the Konica Minolta SPAD-502Plus), NDVI (NDV) (using the GreenSeeker handheld to instantly take a reading of your crop’s health), plant Height (HP), leaf number (LN), stem diameter (SD), diameter of the ear (OED), node number (NN), weight of ear (WE), weight of cob corn (WC), number of seeds per row (NSR), number of seeds per column (NSC), length of ear (LE), weight of seeds per ear (WSE), number of seeds per ear (NSE), weight of fresh plant (WFP), weight of 1000 seeds (1S), and grain yield (GY). Chlorophyll content (Chl) is the green pigment found in the leaves of green plants. The amount of chlorophyll is a good sign of plant health in the leaves, and it is approximately directly proportionate to the amount of nitrogen in the leaves. Leaf chlorophyll concentration is an important parameter that is often measured as an indicator of chloroplast growth, photosynthetic capacity, leaf nitrogen content, or general plant health. Chlorophyll was measured using a SPAD device and NDVI using the GreenSeeker five times during the vegetative period. The other traits were measured during harvesting time. NDVI provides information on the state of a plant’s health by analyzing the absorption and reflection values of red and infrared light. SPAD is a tool for the measurement of chlorophyll [24]. Plant height is an essential variety of attributes. It was found that the tallest hybrids had the greatest genetic distance between the parental components [25]. Corn is susceptible to leaves, and if the number of leaves (LN) used is low, the production factors will not be optimized [26]. Wajid et al. [27] reported that increasing fertilizer increases plant height (HP) and the number of nodes (NN). Increasing the fertilizer amount caused increased stem diameter (SD) but was not significant in the difference between the two hybrids [28]. The number of seeds per column (NSC) and per row (NSR) had an effective fertilizer and environmental condition [29]. The number of the seeds per ear (NSE) is one of the essential performance components on maize, which affects all the elements of nutrients and soil moisture [30]. The weight of seeds per ear (WSE) regulates maize yield, but it is less sensitive than other yield components [31]. Weight of 1000 seeds (1S) is one of the vital grain yield components. No adverse environmental conditions and optimal plant nutrition will be more affected by the genotype [4]. Weight of cob corn (WC) and ear (WE) form in the middle of the plant. The distance and proximity of the leaves to the ear effectively provide the photosynthetic material needed to fill the seed [24]. Outer ear diameter (OED) is an essential element to component yield [24]. With the use of long length of ear (LE) and the number of seeds per row in the correction of maize and the production, suitable compounds are helpful and cause the stability of maize hybrids produced [30]. The grain yield (GY) and weight of fresh plant (WFP) were most correlated with the number of grains in-ear and grains per row.

In the AMMI model, differences between stability and compatibility of genotypes in different environments can be evaluated qualitatively using biplot charts. BIPLOT AMMI is a graph on which environments and genotypes are plotted simultaneously, and their interface will be graphically visible. Therefore, in a biplot, the characteristics of the genotype and the environment should be different. More than two AMMI biplots (AMMI2 and AMMI) were used to check the stability of genotypes. In the AMMI model, only IPCA1 was used to check the stability of genotypes. In the AMMI1 model’s plotted plot, the horizontal axis is the mean performance axis, and the vertical axis is IPCA1. In the AMMI2 model, two components, IPCA1 and IPCA2, were used to evaluate genotypes’ stability and plotted with the AMMI2 model plot [32]. In the GGE biplot graphical process, choices are made on the basis of graphical data investigation and data, unlike conventional methods. This technique includes numerous capacities and clarity in interpreting results. In this way, the evaluations are based on graphic images, not based on outputs generated in tables, etc. The GGE biplot model of Yan et al. [15] has attracted quantitative, biomedical, and racial geneticists for its ease of analysis and evaluation [11]. The GGE biplot emphasizes two concepts: (1) Although the measured yield is a combination of genotype, environment, and genotype–environment interaction, as stated above, only genotype–effect and genotype–environment interactions should consider evaluation in the cultivar or genotype simultaneously. For this reason, the first part of this method is called GGE (GE + G). (2) The Biplot method developed by Gabriel [33] was used to represent the GGE in-field performance tests. For this reason, this method became known as the GGE biplot method [11]. The SPSS, Gen stat, and Minitab were software used for analysis of data.

## 3. Results

### 3.1. Compound Variance Analysis on Traits in FAO410 and FAO340 Hybrids

Combined variance analysis indicated in FAO410 and FAO340 hybrids that the effect of NPK fertilizer was significant for chlorophyll, NDVI, plant height, leaf number, stem diameter, the diameter of the ear, node number, ear weight, weight of cob corn, number of seeds per column, length of ear, weight of seeds per ear, grain in-ear number, weight of fresh plant, weight of 1000 seeds, and grain yield at 1%. Thus, there was variety in the mentioned traits. Genotype effect significant for chlorophyll, the weight of ear, weight of cob corn, length of ear, 1000 grain weight at 1%, leaf number, and the weight of grain ear at 5%. The mentioned traits had variety in terms of genotype. The year was significant in all traits except the number of seeds per row. Genotype by NPK fertilizer interaction effect was significant with regards to the grain in-ear number, the number of seeds per column, and grain in-ear number at 1%. Thus, genotypes by different NPK fertilizer levels had various traits: grain in-ear number, the number of seeds per column, and weight of ear. In the terms of year of NPK interaction effect, all traits were significant at 1%, except plant height, number of seed per row, and the number of seeds per column. The year in genotype interaction effect indicated that NDVI, weight of cob corn, the weight of ear, number of seeds per column, the weight of fresh plant, the weight of 1000 seeds, and grain yield were significant at 1%, and nodes number, weight of seeds per ear, and grain in-ear number at 5%. Therefore, genotypes varied in the mentioned traits per year. The genotype by traits per year in NPK fertilizer interaction effect showed that the weight of cob and weight of 1000 seeds was significant at the level of 1%, and the weight of ear, number of seeds per row, number of seeds per column, length of ear, and weight of seeds per ear at the level of 5%. Thus, genotypes had variations at different NPK fertilizer levels in terms of weight of cob corn, the weight of 1000 seeds, the weight of ear, number of seeds per row, number of seeds per column, length of ear, and weight of seeds per ear (Table 1).

### 3.2. Correlation Analysis on Traits in FAO410 and FAO340 Hybrids

A positive correlation was found between chlorophyll with (NDVI, weight of ear, weight of cob corn, number of seeds per column, length of ear, all seed per weight of ear, and grain yield), plant height with (nodes number, weight of ear, weight of cob corn, number of seeds per column, length of ear, weight of all seeds per ear, grain in ear number, and grain yield), leaf number with (nodes number and grain yield), stem diameter with diameter of the ear, weight of ear with (weight of cob corn, number of seeds per column, length of ear, weight of all seeds per ear, grain in ear number, weight of 1000 seeds, and grain yield), weight of cob corn with (number of seeds per column, length of ear, weight of all seeds per ear, grain in ear number, and grain yield), number of seeds per column with (ear length, the weight of all seed per ear, grain in ear number, and grain yield), weight of all seeds per ear with (weight of 1000 seeds and grain yield), and weight of 1000 seeds with grain yield in the FA O410 hybrid. Moreover, a negative correlation existed between stem diameter with (weight of cob corn and length of ear) and diameter of the ear with (weight of cob corn and length of ear). Correlation analysis showed that positive correlation existed between chlorophyll with (weight of ear, weight of cob corn, length of ear, weight of all seeds per ear, grain in ear number, the weight of fresh plant, and grain yield), plant height with (leaf number, weight of cob corn, weight of all seeds per ear, and grain yield), leaf number with nodes number, stem diameter with diameter of ear, weight of ear with (weight of cob corn, length of ear, grain in ear number, weight of fresh plant, weight of 1000 seeds, and grain yield), weight of cob corn with (length of ear, all seed per weight of ear, weight of fresh plant, weight of 1000 seeds, and grain yield), number of seeds per column with (weight of all seeds per ear, grain in ear number, and grain yield), length of ear with (grain in ear number and the fresh plant in hectare weight), weight of all seeds per ear (weight of fresh plant, weight of 1000 seeds, and grain yield), grain in ear number with (weight of fresh plant and grain yield), weight of fresh plant with (weight of 1000 seeds and grain yield), and 1000 grains with grain yield in FAO340 hybrid. Moreover, there was a negative correlation between stem diameter with (weight of cob corn, length of ear, weight of fresh plant, and weight of 1000 seeds) and the diameter of the ear with (weight of cob corn, length of ear, weight of fresh plant, and weight of 1000 seeds) (Table 2).

### 3.3. Additive Main Effects and Multiplicative Interaction on Traits in FAO410 and FAO340 Hybrids

**Figure 2:**
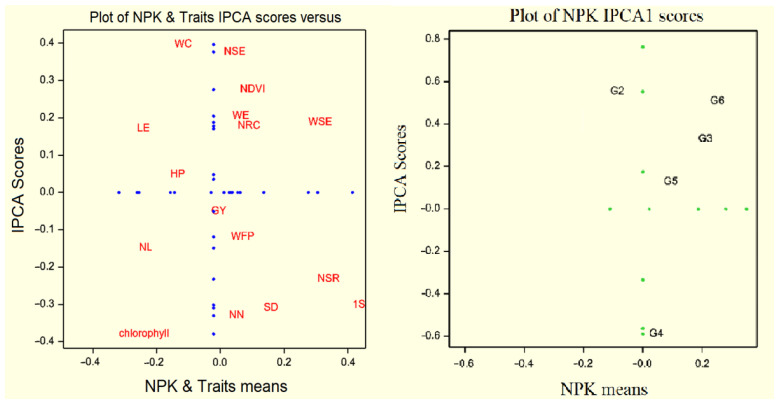
Biplot average traits and NPK of hybrid FAO410 on different treatment levels at principal component values (AMMI). Chlorophyll (Chl), NDVI (NDV), plant height (HP), leaf number (LN), stem diameter (SD), diameter of ear (OED), node number (NN), weight of ear (WE), weight of cob corn (WC), weight of ear (WE), weight of cob (WC), number of seeds in row (NSR), number of seeds per column (NSC), length of ear (LE), weight of all seeds in ear (WSE), number of seeds per ear (NSE), weight of fresh plant (WFP), weight of 1000 seeds (1S), grain yield (GY). G0 until G5 treatments NPK.

**Figure 3:**
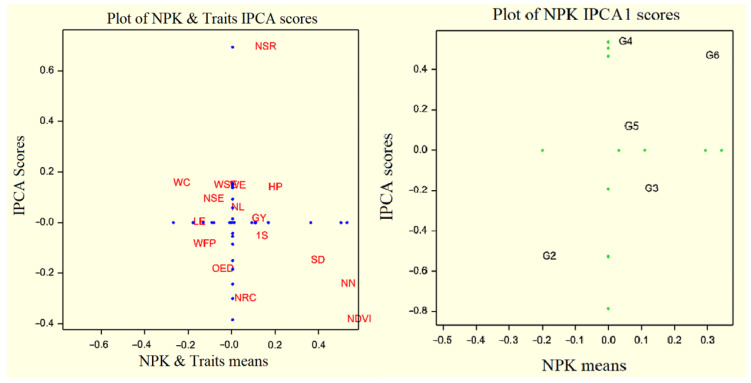
Biplot average traits and NPK of hybrid FAO340 on different treatment levels at principal component values (AMMI). Chlorophyll (Chl), NDVI (NDV), plant height (HP), leaf number (LN), stem diameter (SD), diameter of ear (OED), node number (NN), weight of ear (WE), weight of cob corn (WC), weight of ear (WE), weight of cob (WC), number of seeds in row (NSR), number of seeds per column (NSC), length of ear (LE), weight of all seeds in ear (WSE), number of seeds per ear (NSE), weight of fresh plant (WFP), weight of 1000 seeds (1S), grain yield (GY). G0untilG5 treatments NPK.

To investigate genotype by trait interaction, we applied AMMI model principal component analysis in FAO410 and FAO340 hybrids, explaining significance at 1% effect of the first principal component. Therefore, the AMMI model is considered one main component. In this study, AMMI was used to evaluate traits’ stability, as presented in Table 3 and Table 4. The genotype by trait interaction shows that the first principal component effect was significant at 1%. The first principal component showed 54.24%, and the second principal component, 20.75%, explained the total squares interaction using the AMMI model in the FAO410 hybrid (Table 3). AMMI biplot can evaluate the treatments with trait interaction effect. Traits had the highest interaction between different fertilizer treatments: grain yield, plant height, weight of fresh plant, and leaf number, which had a maximum effect on the FAO410 hybrid’s performance. Therefore, these traits had desirable stability on different fertilizer treatments to the FAO410 hybrid. On the other hand, chlorophyll had the minimum stability of this hybrid on different NPK fertilizer treatments. Desirable treatments are stability, and adaptability includes NPK4, NPK2, and NPK5 (Figure 2). In the FAO340 hybrid, the first principal component showed 58.18%, and the second principal component, 18.04%, explaining the total squares interaction by using the AMMI model in FAO340 hybrid (Table 4). Moreover, traits that had the highest interaction between different fertilizer treatments included leaf number, plant height, weight of fresh plant, and weight of 1000 grains, which had the maximum effect on the FAO340 hybrid’s performance. Thus, these traits had desirable stability on different fertilizer treatments to the FAO340 hybrid. On the other hand, NDVI and the number of nodes had minimum stability of this hybrid in different NPK fertilizer treatments. Desirable treatments are stability, and adaptability includes NPK4, NPK5, and NPK3 (Figure 3).

### 3.4. GGE Biplot Graphical Method Analysis on Traits in FAO410 and FAO340 Hybrids

**Figure 4:**
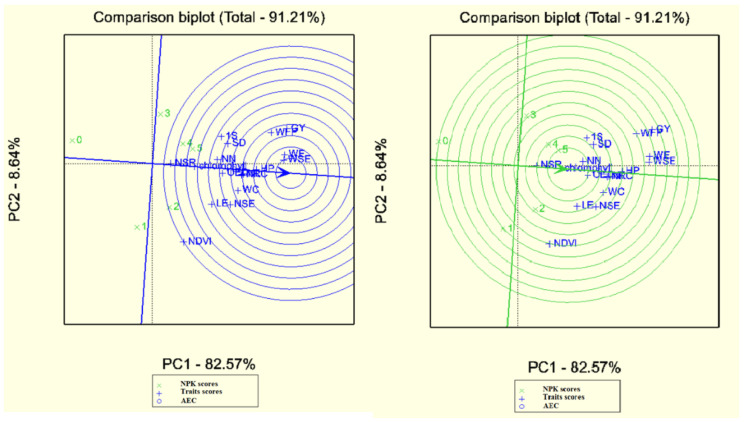
Determination of ideal traits and treatments with GGE biplot on FAO410. Chlorophyll (Chl), NDVI (NDV), plant height (HP), leaf number (LN), stem diameter (SD), diameter of ear (OED), node number (NN), weight of ear (WE), weight of cob corn (WC), weight of ear (WE), weight of cob (WC), number of seeds in row (NSR), number of seeds per column (NSC), length of ear (LE), weight of all seeds in ear (WSE), number of seeds per ear (NSE), weight of fresh plant (WFP), weight of 1000 seeds (1S), grain yield (GY). 0until5 treatments NPK.

**Figure 5:**
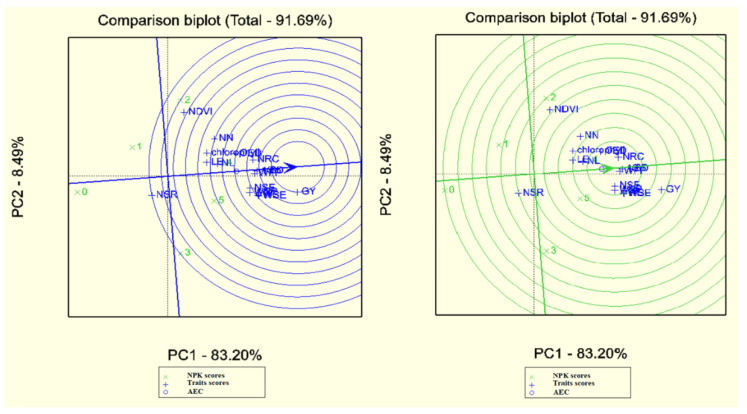
Determination of ideal traits and treatments with GGE biplot on FAO410. Chlorophyll (Chl), NDVI (NDV), plant height (HP), leaf number (LN), stem diameter (SD), diameter of ear (OED), node number (NN), weight of ear (WE), weight of cob corn (WC), weight of ear (WE), weight of cob (WC), number of seeds in row (NSR), number of seeds per column (NSC), length of ear (LE), weight of all seeds in ear (WSE), number of seeds per ear (NSE), weight of fresh plant (WFP), weight of 1000 seeds (1S), grain yield (GY). 0until5 treatments NPK.

Desirable traits are the most recognizable and representative of other traits. Accordingly, the weight of seeds per ear and weight of ear were recognized as desirable attributes due to proximity to the middle of concentric circles. Finally, the number of seeds per row and NDVI were introduced as the most powerless attributes due to their greater length from the center of concentric circles. It must be commented that the excellent attribute is a desirable representative for the study of treatments (although this is not a cause to deny the results of other attributes). The desirable attribute means the most desirable routine of treatment response in the FAO410 hybrid. The first and second principal components covered 91.20% of this analysis’s total data (Figure 3). One of the main usages of biplot is identifying the best treatments according to various measured indicators or traits. This indicates the treatments’ ranking according to the desirable treatment regarding treatments tending to move towards to the positive end of the treatments’ mean axis and vertical distance. The similarity and proximity of the desirable treatments and the appropriate treatment can be easily identified. Accordingly, the desirable treatment was NPK5, followed by NPK4, NPK2, NPK3, NPK1, and NPK0. NPK4 and NPK5 were the most desirable treatments for the number of seeds per row, chlorophyll, weight of 1000 grains, and stem diameter in FAO410 hybrid (Figure 4). The grain yield, the fresh plant’s weight, stem diameter, and the weight of 1000 seeds were recognized as desirable traits due to their proximity to the center of concentric circles. NDVI and the number of seeds per row were introduced as invalid traits due to their greater level of space from the center of concentric circles. The first and second principal components covered 91.69% of the total data in this FAO340 hybrid (Figure 4). The desirable treatment was NPK4, followed by NPK5, NPK2, NPK3, NPK1, and NPK0. The leaf number and length of the ear were the most desirable in NPK5 and NPK4 in the FAO340 hybrid (Figure 5).

## 4. Discussion

The significance of the effects means that the NDVI had a variety of these effects. NDVI was positive on the first factor and negative on the second factor in factor analysis. In general, NDVI had minimum desirability and stability in FAO410 and FAO340 hybrids. The significance of the effects means that the chlorophyll had a variety of these effects. Chlorophyll was positive on the first and second factors in PCA analysis. In general, chlorophyll had a positive correlation with plant height, weight of ear, weight of cob corn, number of seeds per column, length of ear, weight of seeds per ear, and grain yield in FAO410 hybrid. Chlorophyll had a positive correlation between weight of cob corn, length of ear, weight of seeds per ear, grain in-ear number, and grain yield in FAO340 hybrid. The positive effect of fertilizer application on plant yield has been proven in many experiments [34]. Nitrogen causes increased plant yield by increasing leaf area index and chlorophyll content. Nitrogen, potassium, and phosphorus are critical factors in achieving optimal corn yield. This plant requires specific nutrients in the relatively short growing period, one of the most critical factors in maintaining photosynthetic capacity. Decreased photosynthesis appears to be partly due to a decrease in chlorophyll [23]. There was a positive correlation between plant height with leaf number, chlorophyll, node number, weight of ear, weight of cob corn, number of seeds per column, weight of seeds per ear, grain in-ear number, and seed grain yield in FAO410 hybrid. Leaf number was an essential factor in the first factor in FAO410 and the second factor for FAO340 in factor analysis. Leaf number had a positive correlation with the number of nodes and grain yield in FAO410, and a positive correlation with the number of nodes in FAO340. Leaf number was positive in the first principal component and negative in the second principal component in FAO410 and FAO340. AMMI analysis showed that the leaf number had desirable stability in FAO340. Stem diameter was a positive correlation with the outer ear diameter at hybrids. Moreover, it negatively correlated with the weight of cob and length of ear in FAO410 and had a negative correlation with weight of cob, length of ear, weight of the fresh plant, and weight of 1000 seeds in FAO340. In many studies, fertilizer’s positive effect emphasized raising grain yield, the number of kernels in an ear, and kernel value in different corn genotypes. Fertilizer causes proliferation of the leaf number, leaf size and width, and the plant’s dry matter [35,36,37,38,39,40,41]. The number of seeds per row had a minimum effect for treatments and low stability in terms of GGE analysis. The compound analysis showed that existed significant effect of NPK; year; NPK and year interaction; genotype and year interaction; and NPK, genotype, and year interaction in the hybrids. The number of seeds per column had a positive correlation with length of ear, the weight of seeds per ear, the number of seeds per ear, grain yield, chlorophyll, plant height, the weight of cob, and weight of ear in FAO410. Moreover, it had a positive correlation with the weight of all seeds per ear, the number of seeds per ear, and grain yield in FAO340. The grain in-ear number had a favorable correlation with height of corn, weight of cob corn, weight of ear, and number of seeds per column in FAO410, and grain in-ear number with weight of fresh plant, grain yield, chlorophyll, weight of ear, number of seeds per column, size of the ear, and weight of seeds per ear in FAO340. The number of seeds per ear had desirable stability on FAO340 by AMMI analysis. The weight of 1000 seeds was not correlated with the length of the ear and the number of grains in the row [42], whereas we reported a positive and significant correlation between ear lengths, the weight of 1000 seeds, and grain yield. A positive correlation was reported between grain yield and the number of seeds per row [41,43]. Weight of all seeds per ear had a positive correlation with weight of 1000 grains, grain yield, chlorophyll, plant height, the weight of ear, weight of cob corn, and grain in-ear number in FAO410, and also had a positive correlation with the number of seeds per ear, the weight of the fresh plant, the weight of 1000 seeds, grain yield, chlorophyll, plant height, the weight of cob, the weight of ear, and a number of seeds per column in FAO340. Weight of all seeds per ear had desirable stability and maximum effect on FAO340 by AMMI analysis and a significant effect on hybrids by GGE analysis. Weight of 1000 seeds had a positive correlation with grain yield, the weight of ear, and weight of seeds per ear on FAO410, as well as a positive correlation with grain yield, the diameter of the ear, the weight of ear, weight of cob corn, weight of fresh plant, and grain in-ear number on FAO340.

The weight of all seeds per ear had desirable stability on FAO340 by AMMI analysis. Weight of cob corn positively correlated with number of seeds per column, length of ear, the weight of seeds per ear, grain yield, chlorophyll, plant height, and weight of ear on FAO410. Moreover, it had a positive correlation with length of ear, weight of seeds per ear, weight of fresh plant, weight of 1000 grains, grain yield, chlorophyll, plant height, and weight of ear. The weight of 1000 seeds in corn is a function of the plant’s ability to provide nutrients for reservoirs and environmental conditions such as the availability of moisture and nutrients during the filling seed stage [44]. The NPK treatments significant on grain yield, biological yield, harvest index, ear yield, ear length and diameter, weight of ear, number of seeds per ear, and weight of 1000 seeds. The yield of late corn hybrids concluded that the number of seeds in a row and the weight of 1000 seeds had a positive and significant correlation with grain yield [45]. Weight of ear had a positive correlation with weight of cob corn, number of seeds per column, the length of ear, grain in-ear number, weight of 1000 grains, grain yield, chlorophyll, and plant height in FAO410, and had a positive correlation with weight of cob corn, length of ear, weight of seeds per ear, grain in-ear number, weight of fresh plant, grain yield, and chlorophyll on FAO340. Weight of ear was desirable in grain yield by AMMI and GGE analysis on hybrids. Correlation analysis showed that the diameter of the ear negatively correlated with weight of cob corn and length of ear and had a positive correlation with stem diameter on FAO410. A negative correlation with weight of cob corn, length of ear, weight of fresh plant, weight of 1000 seeds and a positive correlation with stem diameter was found for FAO340. The significance of the effects means that the fresh plant’s weight had a variety of these effects. Factor analysis indicated that weight of all seeds per ear was in the third group in FAO410 and was in the first group in FAO340. Weight of fresh plant had a positive correlation with weight of 1000 grains, grain yield, chlorophyll, the weight of cob corn, the ear weight, length of ear, weight of seeds per ear, and the number of grains per ear on FAO340. Moreover, it had a negative correlation with the diameter of the ear and stem diameter. The weight of fresh plants had desirable stability and a maximum effect on hybrids’ performance by AMMI analysis. The weight of fresh plants had a maximum effect on grain yield by GGE analysis. Grain yield positively correlated with chlorophyll, plant height, leaf number, ear weight, weight of cob corn, number of seeds per column, weight of seeds per ear, and weight of 1000 seeds on FAO410. Grain yield had desirable stability and maximum effect by AMMI analysis on genotypes. GGE analysis showed that grain yield and the fresh plant’s weight had a maximum effect on NPK fertilizer levels on genotypes. Using complete fertilizer can generate maximum grain yield in maize, but using potassium and phosphorus with nitrogen can stabilize grain yield and other parameters. Therefore, using the complete fertilizer NPK dose with 150 Kg/ha nitrogen, 115 Kg/ha potassium, and 135 Kg/ha phosphorus can improve yield and yield components according to sustainable agriculture on maize hybrids [46]. Akbari et al. [47] showed that increasing the nitrogen amount of fertilizers and potassium increased corn’s weight. However, the use of nitrogen fertilizer alone, even in large quantities, could not positively affect the weight of 100 grains. According to the results of step-by-step regression, the physiological traits, plant height, grain depth, number of seeds per row, and length of ear explained a total of 68.60% of the variation related to yield [48]. It was observed that the consumption of 240 kg of fertilizer per hectare in corn increased the speed of leaf emergence and grain yield [49]. The grain yield was mostly correlated with the number of grains in-ear and the number of seeds per row. In the last sample of multiple regression analysis, the remaining traits included the weight of 100 grains, the number of grains in-ear, the number of leaves, and the height of the plant [50].

## 5. Conclusions

Increasing NPK consumption can lead to better plant physiological conditions due to nutrient uptake and more favorable environmental and food conditions than adequate access. In plant breeding, the selection is based on many agronomic traits that may negatively or positively correlate. Therefore, analysis methods that reduce significant performance traits without eliminating large amounts of helpful information are valuable to researchers. Traits that had the highest interaction between different fertilizer treatments included: grain yield, plant height, weight of fresh plant, and number of leaves, having the maximum effect on the FAO410 hybrid’s performance. Therefore, these traits had desirable stability on different fertilizer treatments to FAO410 hybrid. Moreover, traits that had the highest interaction between different fertilizer treatments included leaf number, plant height, weight of fresh plant, and weight of 1000 seeds, having the maximum effect on the FAO340 hybrid’s performance. Therefore, these traits had desirable stability on different fertilizer treatments to FAO340 hybrid. In this regard, the use of correlations between traits is common. Knowing how different traits relate is essential in developing breeding programs to increase grain yield because one-way selection for crop traits without correlation with other traits will not result. Therefore, the correlation between traits should be considered in breeding programs. In sustainable agriculture, fertilizers have a special role in increasing crop production and maintaining sustainable soil fertility. The effect of fertilizers on yield components and knowing their effect helps to maintain balance and stability in chemical fertilizers. This research showed that the weight of cob, plant height, the weight of 1000 seeds, and the number of leaves had important factors to obtain optimized fertilizer on the basis of sustainable agricultural on maize hybrids.

## Figures and Tables

**Table 1:** Compound variance analysis on grain yield in FAO410 and FAO340 hybrids in six NPK treatments.

S.O.V.	DF	Chl	NDV	HP	LN	SD	OED	NN	WE	WC	NSR	NSC	LE	WSE	NSE	WFP	1S	GY
NPK	5	26.12 **	66.88 **	13.85 **	12.38 **	24.97 **	24.97 **	8.82 **	95.36 **	51.92 **	0.82	21.09 **	8.72 **	98.05 **	23.06 **	56.69 **	33.69 **	190.24 **
Genotype	1	31.25 **	2.02	0.039	4.16 *	0.43	0.43	1.05	9.99 **	67.86 **	0.76	0.01	5.62 **	3.93 *	0.29	741.65	12.97 **	2.37
Year	2	51.38 **	41.78 **	6.11 **	10,677.6 **	59,320.5 **	59,320.5 **	10.54 **	97.24 **	322.69 **	2.58	33.57 **	94.01 **	45.52 **	6.19 **	557.15 **	64.39 **	75.71 **
NPK * Genotype	5	0.67	0.91	1.15	0.73	0.74	0.74	0.31	2.39 *	1.85	0.88	3.16 *	0.62	2.02	2.62 *	1.05	1.76	0.30
NPK * Year	10	10.45 **	37.83 **	1.58	4.02 **	10.36 **	10.36 **	2.19 *	6.71 **	6.57 **	1.47	1.99	4.86 **	5.78 **	2.95 **	7.31 **	8.15 **	3.74 **
Rep * NPK * Year	45	1.33	10.21 **	1.33	0.88	1.47	1.47	1.45	1.50	1.51	1.59	1.88 *	1.01	1.48	1.95 **	1.01	1.35	0.83
Genotype * Year	2	0.04	20.68 **	2.59	23.03	2.47	2.47	3.37 *	5.97 **	14.11 **	2.73	9.78 **	0.86	4.34 *	3.51 *	722.32 **	33.55 **	5.22 **
Year * Genotype * NPK	10	0.67	0.79	0.96	1.08	0.69	0.69	0.39	2.61 *	6.17 **	2.25 *	2.52 *	2.14 *	2.05 *	1.27	1.05	4.25 **	1.53
Error	54	0.024	0.005	0.005	0.011	0.005	0.005	0.006	0.436	0.068	0.01	0.074	0.004	0.432	1.781	10,812	0.347	17,291

Chlorophyll (Chl), NDVI (NDV), plant height (HP), leaf number (LN), stem diameter (SD), diameter of ear (OED), node number (NN), weight of ear (WE), weight of cob corn (WC), weight of ear (WE), weight of cob (WC), number of seeds in row (NSR), number of seeds per column (NSC), length of ear (LE), weight of all seeds in ear (WSE), number of seeds per ear (NSE), weight of fresh plant (WFP), weight of 1000 seeds (1S), grain yield (GY); ** and * indicate significance at 1% and 5% percent.

**Table 2:** Correlation analysis of FAO410 and FAO340 in six NPK treatments.

		Chl	NDVI	HP	LN	SD	OD	NN	WE	WC	NSR	NSC	LE	WSE	NSE	WFP	1S
FAO410	NDVI	0.324															
	HP	0.687	0.335														
	LN	0.417	0.362	0.586													
	SD	−0.380	0.413	−0.308	−0.004												
	OD	−0.399	0.403	−0.332	−0.020	0.996											
	NN	0.412	0.346	0.530	0.836	−0.114	−0.132										
	WE	0.637	0.229	0.656	0.458	−0.349	−0.358	0.367									
	WC	0.741	0.046	0.671	0.314	−0.747	−0.764	0.346	0.736								
	NSR	−0.147	−0.024	0.017	0.047	0.076	0.099	−0.040	−0.002	−0.141							
	NSC	0.586	0.242	0.690	0.405	−0.407	−0.436	0.421	0.567	0.698	−0.193						
	LE	0.622	0.059	0.732	0.293	−0.576	−0.598	0.336	0.505	0.748	−0.208	0.673					
	WSE	0.622	0.301	0.632	0.481	−0.177	−0.190	0.337	0.926	0.604ta	−0.026	0.514	0.390				
	NSE	0.413	0.375	0.597	0.397	−0.176	−0.191	0.351	0.504	0.514	0.197	0.826	0.482	0.481			
	WFP	0.011	0.207	0.229	0.207	0.317	0.281	0.236	−0.120	−0.074	−0.175	0.248	0.179	−0.101	0.206		
	1S	0.332	0.268	0.214	0.268	0.060	0.062	0.209	0.596	0.165	−0.004	0.106	0.059	0.605	0.052	−0.345	
	GR	0.654	0.319	0.644	0.521	−0.196	−0.211	0.371	0.918	0.627	−0.059	0.538	0.399	0.973	0.458	−0.110	0.63
FAO340	NDVI	0.323															
	HP	0.244	0.303														
	LN	0.219	0.275	0.527													
	SD	−0.43	0.122	−0.084	−0.009												
	OD	−0.44	0.109	−0.126	−0.063	0.995											
	NN	0.191	0.342	0.487	0.856	0.108	0.056										
	WE	0.511	0.352	0.475	0.297	−0.438	−0.462	0.294									
	WC	0.528	0.281	0.519	0.353	−0.580	−0.609	0.346	0.893								
	NSR	0.216	0.204	0.144	0.002	−0.210	−0.204	−0.129	0.199	0.180							
	NSC	0.476	0.312	0.331	0.196	−0.243	−0.265	0.202	0.487	0.426	−0.095						
	LE	0.543	0.249	0.303	0.329	−0.503	−0.527	0.344	0.566	0.724	0.087	0.441					
	WSE	0.555	0.374	0.538	0.371	−0.294	−0.317	0.330	0.880	0.745	0.252	0.522	0.465				
	NSE	0.513	0.483	0.360	0.241	−0.089	−0.113	0.205	0.581	0.454	0.396	0.652	0.530	0.642			
	WFP	0.642	0.255	0.359	0.271	−0.568	−0.584	0.236	0.688	0.696	0.313	0.499	0.656	0.688	0.591		
	1S	0.391	0.182	0.249	0.267	−0.503	−0.513	0.160	0.753	0.671	0.031	0.439	0.371	0.711	0.267	0.570	
	GR	0.567	0.397	0.543	0.360	−0.288	−0.311	0.317	0.854	0.711	0.280	0.507	0.435	0.981	0.641	0.689	0.686

Chlorophyll (Chl), NDVI (NDV), plant height (HP), leaf number (LN), stem diameter (SD), diameter of ear (OED), node number (NN), weight of ear (WE), weight of cob corn (WC), weight of ear (WE), weight of cob (WC), number of seeds in row (NSR), number of seeds per column (NSC), length of ear (LE), weight of all seeds in ear (WSE), number of seeds per ear (NSE), weight of fresh plant (WFP), weight of 1000 seeds (1S), grain yield (GY).

**Table 3:** Variance analysis by AMMI model FAO410 in six NPK treatments.

S.O.V.	DF	SS	SS%	F
Total	1223	1178		
Treatments	101	221.0		2.57
NPK	5	157.4		36.99
Traits	16	0.0		0.00
Block	51	45.6		1.05
Interactions	80	63.6		0.93
IPCA_1_	20	34.5	54.24	2.03
IPCA_2_	18	13.2	20.75	0.86
Residuals	42	15.9	25.01	0.44
Error	1071	911.4		

**Table 4:** Variance analysis by AMMI model FAO340 in six NPK treatments.

S.O.V.	DF	SS	SS%	F
Total	1223	1186		
Treatments	101	180		2.09
NPK	5	120.8		28.37
Traits	16	0.0		0.00
Block	51	94.1		2.17
Interactions	80	59.3		0.87
IPCA_1_	20	34.5	58.18	2.03
IPCA_2_	18	10.7	18.04	0.70
Residuals	42	14.1	23.78	0.39
Error	1071	911.4		

## Data Availability

All data supporting the conclusions of this article are included in this article.

## Abbreviations

Chlorophyll (Chl), NDVI (NDV), plant height (HP), leaf number (LN), stem diameter (SD), diameter of ear (OED), node number (NN), weight of ear (WE), weight of cob corn (WC), weight of ear (WE), weight of cob (WC), number of seeds in row (NSR), number of seeds per column (NSC), length of ear (LE), weight of all seeds in ear (WSE), number of seeds per ear (NSE), weight of fresh plant (WFP), weight of 1000 seeds (1S), grain yield (GY).
